# In Situ and Ex Situ Designed Hydroxyapatite: Bacterial Cellulose Materials with Biomedical Applications

**DOI:** 10.3390/ma13214793

**Published:** 2020-10-27

**Authors:** Adrian Ionut Nicoara, Alexandra Elena Stoica, Denisa-Ionela Ene, Bogdan Stefan Vasile, Alina Maria Holban, Ionela Andreea Neacsu

**Affiliations:** 1 Department of Science and Engineering of Oxide Materials and Nanomaterials, Faculty of Applied Chemistry and Materials Science, University Politehnica of Bucharest, 060042 Bucharest, Romania; adrian.nicoara@upb.ro (A.I.N.); bogdan.vasile@upb.ro (B.S.V.); ionela.neacsu@upb.ro (I.A.N.); 2 National Research Center for Micro and Nanomaterials, Faculty of Applied Chemistry and Materials Science, University Politehnica of Bucharest, 060042 Bucharest, Romania; 3 Faculty of Engineering in Foreign Languages, University Politehnica of Bucharest, 060042 Bucharest, Romania; denisa_ionela.ene@stud.fim.upb.ro; 4 Microbiology Department, Faculty of Biology, University of Bucharest, 060101 Bucharest, Romania; alina.m.holban@bio.unibuc.ro

**Keywords:** bacterial cellulose, hydroxyapatite, nanoAg, tissue engineering, antimicrobial composite

## Abstract

Hydroxyapatite (HAp) and bacterial cellulose (BC) composite materials represent a promising approach for tissue engineering due to their excellent biocompatibility and bioactivity. This paper presents the synthesis and characterization of two types of materials based on HAp and BC, with antibacterial properties provided by silver nanoparticles (AgNPs). The composite materials were obtained following two routes: (1) HAp was obtained in situ directly in the BC matrix containing different amounts of AgNPs by the coprecipitation method, and (2) HAp was first obtained separately using the coprecipitation method, then combined with BC containing different amounts of AgNPs by ultrasound exposure. The obtained materials were characterized by means of XRD, SEM, and FT-IR, while their antimicrobial effect was evaluated against Gram-negative bacteria (*Escherichia coli*), Gram-positive bacteria (*Staphylococcus aureus*), and yeast (*Candida albicans*). The results demonstrated that the obtained composite materials were characterized by a homogenous porous structure and high water absorption capacity (more than 1000% *w*/*w*). These materials also possessed low degradation rates (<5% in simulated body fluid (SBF) at 37 °C) and considerable antimicrobial effect due to silver nanoparticles (10–70 nm) embedded in the polymer matrix. These properties could be finetuned by adjusting the content of AgNPs and the synthesis route. The samples prepared using the in situ route had a wider porosity range and better homogeneity.

## 1. Introduction

One of the most significant advances in the field of tissue engineering is the development of a porous three-dimensional matrix [1]. In order to act as an optimal bone support, the synthetic matrix must have a series of properties, including biocompatibility, biodegradability, appropriate porosity (similar to the replaced tissue), antimicrobial activity, and production reproducibility [2,3]. In addition to these requirements, it is also recommended that they have mechanical properties similar to natural bone, such as compressive strength, fatigue resistance, and high Young’s modulus [4].

Such characteristics allow cell penetration, vascularization, and adequate nutrient and oxygen diffusion to cells and to the unformed extracellular matrix, which ensures cells viability. The pore size is, in fact, a key element for material efficiency. The pores must be large enough to allow cells to enter and move into the framework of the scaffold, while a small dimension allows the attachment of essential cell number at the same level [5]. Depending on the type of host tissue, all the support materials used in tissue engineering may have a macroporous structure with a particular pore size. For example, researchers suggest a pore size of 200–400 microns is optimal for bone tissue engineering [5,6].

The inorganic phase of the composites designed for bone replacement is usually hydroxyapatite (HAp) [7,8]. HAp is an essential element required for tissue regeneration, with the advantages of great biocompatibility, high plasticity, and remarkable mechanical properties because its chemical and crystalline structure is similar to natural bone apatite [2,9]. It also has an ultrafine structure and a large surface area that is advantageous for cell–biomaterial interactions and has been widely studied in applications for bone engineering [8,10].

For the organic phase of natural bone, replacement with bacterial cellulose (BC) has been attempted [11]. Even though the BC structure is chemically equivalent to plant cellulose (β-D-glucopyranose units linked by β-1,4 glycosidic bonds), it is free of by-products, such as lignin, pectin, hemicellulose, and other constituents of lignocellulosic materials. BC is a biodegradable polymer consisting of nanofibrillar structures, which determine a high specific surface area and a microporous structure. The unique 3D structure of BC is the main reason for its excellent retention and osteoinductivity, properties that make it a highly desirable substitute for collagen extracellular matrix in hard tissue engineering applications [12]. However, insufficient mechanical strength of the polymer restricts its direct application in vivo [13].

Many studies [14,15,16,17] have shown that BC could provide tissue regeneration and substitution, thus being used for bioengineering of hard, cartilaginous, and soft tissues. Bacterial cellulose is widely used as a wound dressing material, and nanomaterials obtained from BC show great antimicrobial properties [18,19,20,21]. BC can be combined with polymeric and nonpolymeric compounds to acquire or enhance antimicrobial, cell adhesion, and proliferation properties [13,22,23,24,25].

Scaffolds embedded with antimicrobial agents, antibiotics, or several forms of silver nanoparticles, which are known antimicrobial agents, are attracting great interest in biomedical research. Metallic silver and silver nanoparticles (AgNPs) have been reported to provide a wide variety of antimicrobial activities [12,26,27,28].

AgNPs are more toxic compared to bulk silver but they have a strong anti-inflammatory impact during tissue healing and can be integrated into composite materials to obtain antibacterial properties [29,30,31]. The human dietary intake of silver, owing to the widespread use of silver compounds, is estimated at 70–90 μg per day [32]. One of the main risk factors in tissue engineering and implant development is microbial infections. Bacterial colonization and the development of multicellular attached communities, called biofilms, are responsible for the high rate of failure in tissue engineering [33].

The purpose of this study was to develop a composite material based on hydroxyapatite, bacterial cellulose, and silver nanoparticles with biomedical applications. The material was obtained by the coprecipitation technique, which is a reliable, simple, economic, fairly rapid, and precise method that allows the synthesis of homogenous structures and favorable pore dimensions [10]. Studies have described the synthesis of bacterial cellulose/hydroxyapatite composites for bone healing applications using different methods [34,35,36,37,38,39]. In this work, AgNPs were integrated in the composite system in order to induce antibacterial properties.

## 2. Materials and Synthesis Methods

The chemical reagents were calcium nitrate tetrahydrate (Ca(NO_3_)_2_•4H_2_O, >99%), ammonium phosphate dibasic ((NH_4_)_2_HPO_4_, 99%), ammonium hydroxide (NH_4_OH, 99%), sodium hydroxide (NaOH, 98%), silver nitrate (AgNO_3_, >99%), sodium citrate (C_6_H_5_O_7_Na, >99%), polyvinylpyrrolidone ((C_6_H_9_NO)_n_), and sodium borohydride (NaBH_4_, >99%), purchased from Sigma-Aldrich (St. Louis, MO, USA). The solvents were American Chemical Society (ACS, Washington, DC, USA) purity. Bacterial cellulose membrane was produced in the laboratory by *Gluconacetobacter* sp. strain isolated from traditionally fermented apple vinegar in the Microbiology Laboratory of the Chemical and Biochemical Engineering Department, University Politehnica of Bucharest, based on a protocol previously described [40].

**Figure 1:**
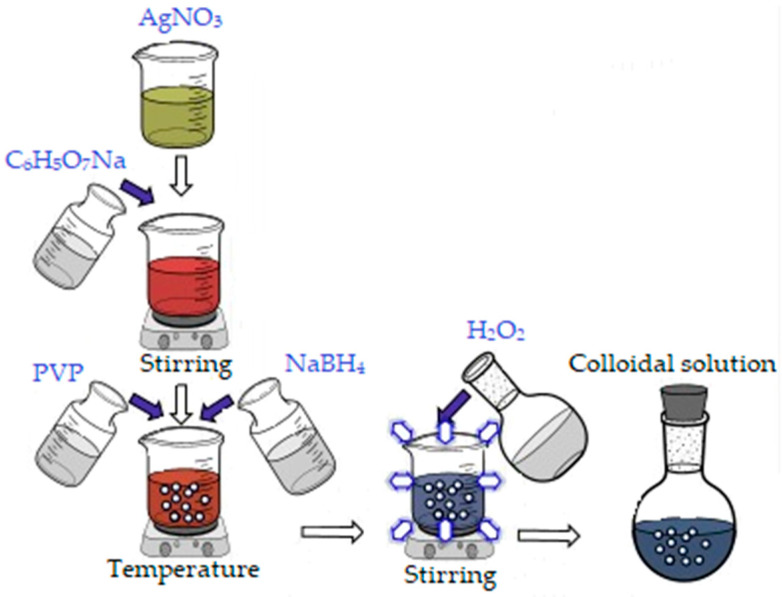
Synthesis of silver nanoparticles.

In order to obtain 500 mL colloidal silver (100 ppm concentration), an aqueous silver nitrate solution (AgNO_3_) was used as silver precursor, to which 30 mL sodium citrate (0.3 M) was added. After 12 min, 30 mL of Polyvinylpyrrolidone (PVP, 0.007 M) and 5 mL NaBH_4_ (1 M) were added to reduce Ag^+^ to Ag^0^ nanoparticles. Finally, 5 mL of oxygenated water (30%) was added, and stirring was maintained for another 10 min approximately, until a light blue color (due to the size of the nanoparticles) was obtained (Figure 1) [41].

The bacterial cellulose synthesized by the Gram-negative bacteria (*Gluconacetobacter* sp.) was boiled at 80 °C in water alkalized with sodium hydroxide (pH 14, measured by colorimetric method). After purification, BC was washed in distilled water until it reached neutral pH. Afterward, it was minced using a blender (Silvercrest, Neckarsulm, Germany) and weighed according to the recipe. Previously, the amount of dry matter was determined on a quantity of BC by eliminating the humidity, and it was found that 0.25 g of dry BC can be obtained from 10.62 g of wet BC [17].

Two synthesis methods were used to obtain the bacterial cellulose and HAp-based composites. The first method involved obtaining in situ hydroxyapatite nanoparticles directly on cellulose fibers and subsequently adding the AgNPs solution, followed by homogenization using an ultrasound probe (composites further referenced as BC_1_, BC_2_, BC_3_, and BC_4_).

For the synthesis of 2 g HAp, the amount of precursors required to obtain the material with different concentrations of AgNPs (0, 1, 2, and 5 wt %) was calculated.

**Figure 2:**
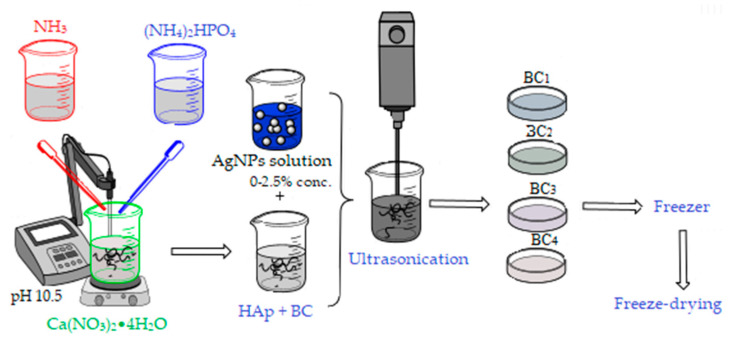
Synthesis of BC_1_, BC_2_, BC_3_, and BC_4_ composites; BC, bacterial cellulose.

The Ca^2+^ and PO_4_^3−^ precursors, Ca(NO_3_)_2_•4H_2_O, and (NH_4_)_2_HPO_4_ were solubilized in distilled water, and bacterial cellulose was added in the calcium nitrate solution (see Figure 2). The mixtures were homogenized by magnetic stirring, and the ammonium phosphate solution was added dropwise. After homogenization, the pH was adjusted to 10.5 with an ammonium hydroxide solution. The obtained precipitates were aged for 24 h, then washed with distilled water until pH 7 was achieved. After washing, the appropriate amount of silver colloidal solution that had been previously obtained was added to each composition according to the centralizing table (Table 1). The obtained mixture was mixed for 3 min in the presence of ultrasound to ensure the best possible homogeneity and then poured into Petri dishes (d = 54 mm), frozen, and subsequently subjected to the freeze-drying process (freezing at −55 °C for 12 h, vacuum at 0.001 mbar for 12 h, and heating under vacuum for 24 h to 35 °C) in order to obtain porous composite materials [42].

**Figure 3:**
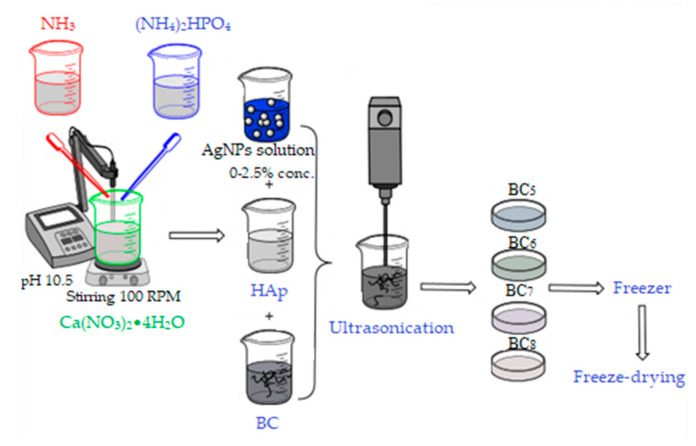
Synthesis of BC_5_, BC_6_, BC_7_, and BC_8_ composites.

The second method of synthesis involved the separate synthesis of HAp by the coprecipitation method, followed by its addition to bacterial cellulose gel in the presence of ultrasound for 3 min, as described in Figure 3.

After homogenization, the required amount of silver colloidal solution was added, followed by the steps previously described in the in situ method. The composites thus obtained by the ex situ method were noted as BC_5_, BC_6_, BC_7_, and BC_8_, and their composition is presented in Table 1.

## 3. Characterization Methods

### 3.1. Physicochemical Characterization

Investigation of the crystallinity of the powders was performed by means of X-ray diffraction (XRD) technique using the PANalytrical Empyrean (Malvern, Bruno, the Netherlands) equipment in Bragg–Brentano geometry equipped with a Cu anode (λCuKα = 1.541874 Å) X-ray tube. The spectra were acquired in the range of 10–80° 2θ angles (Bragg angle) with an acquisition step of 0.02° and an acquisition time of 100 s. The scanning electron microscopy (SEM) images were performed with a FEI Inspect F50 microscope coupled with an energy-dispersive spectrometer (EDS) (FEI, Eindhoven, the Netherlands). Both secondary electron and backscattered electron detectors were used at 30 kV accelerating voltage. The TEM images of AgNPs were obtained using the high-resolution transmission electron microscope TecnaiTM G2 F30 S-TWIN equipped with selected-area electron diffraction (SAED) detector, purchased from the company FEI. This microscope operates in transmission mode at a voltage of 300 kV with a resolution of 2 Å. Research conducted by Fourier transform infrared spectroscopy (FT-IR) involved the analysis of a small amount of samples using the Nicolet iS50R spectrometer (Thermo Fisher Waltham, MA, USA). The measurements were performed at room temperature utilizing the total reflection attenuation module (ATR), and 32 scans of the samples between 4000 and 400 cm^−1^ were performed using a resolution of 4 cm^−1^. The differential thermal analysis (ATD-DSC) were performed using a Shimadzu DTG-TA-50H equipment (Shimadsu, Sanjo, Japan) at 25–700 °C with a heating rate of 10 °C/min.

The open porosity of the freeze-dried composite materials was calculated with Equation (1) for each material prepared in order to observe the porosity level according to the chosen manufacturing method, while the water absorption was calculated with Equation (2):

(1) $$\mathrm{Open}\;\mathrm{porosity}\;(\%)=\frac{M_{we}-M_{d}}{M_{we}-M_{w}}\times 100$$

(2) $$\mathrm{Water}\;\mathrm{absorption}\;(\%)=\frac{M_{we}-M_{d}}{M_{d}}\times 100$$

where *M_we_* is the wet sample weight, *M_d_* is the dry sample weight, and *M_w_* is the sample weight in water.

### 3.2. Degradability

To test their biodegradability, the samples were placed in a 12-well plate in which phosphate-buffered saline (PBS) and simulated body fluid (SBF) were added, similar to the processes involved in the human body. After immersion of the samples in fluid, their integrity was monitored for 7 days. The degradation rate was calculated with Equation (3) for each material:

(3) $$\mathrm{Degradation}\;(\%)=\frac{M_{7day}-M_{initial}}{M_{initial}}\times 100$$

where *M*_7*days*_ is the sample weight in SBF after 7 days of immersion in SBF, and *M_initial_* is the sample weight after immersion in SBF. All the weight values were obtained at room temperature using a hydrostatic analytic balance.

### 3.3. Antimicrobial Efficiency

The antimicrobial behavior of the freeze-dried composite materials was qualitatively assessed by an adapted growth inhibition assay [43]. To cover a wide spectrum of clinically relevant model microbial species, one Gram-positive (*Staphylococcus aureus* ATCC 23235), one Gram-negative bacteria (*Escherichia coli* ATCC 25922), and one yeast (*Candida albicans* ATCC 10231) laboratory strain were used. The standard work protocol for the adapted version of the disc diffusion method implies the preparation of microbial suspensions of 0.5 McFarland standard density (1.5 × 10^8^ colony forming units (CFU)/mL), prepared in sterile buffered saline solution. The obtained microbial suspensions were afterward used to swab inoculate the entire surface of the nutrient agar Petri dishes. After inoculation, identical size samples of the sterile coatings were aseptically placed on the inoculated agar surface, and the plates were incubated at 37 °C for 24 h to allow the growth of bacteria. After incubation, the growth inhibition zone diameter (mm) was measured. A wider inhibition zone suggests a higher antimicrobial effect of the fibrous dressing, reflecting the ability of AgNPs contained into the composite material to diffuse within the agar.

## 4. Results and Discussions

**Figure 4:**
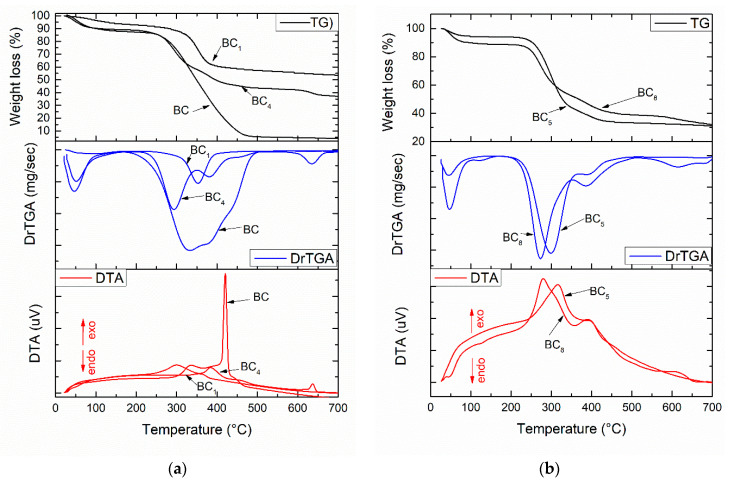
Thermal analysis corresponding to the composite samples: (**a**) BC_1_ and BC_4_; (**b**) BC_5_ and BC_8._

The thermal analysis corresponding to the composite samples are presented in Figure 4. The two minor weight losses that occurred at temperatures below 200 °C were probably related to the volatilization of solvents and physical water. The main mass loss was observed in the range 250–450 °C, with the corresponding exothermic effect being strong and intense and indicating burning of the organic component of the composite (bacterial cellulose). Regarding the compositional aspects, the thermal analysis allowed an accurate assessment of the loading degree depending on the material deposited on the surface or between the fibers of the bacterial cellulose. It was observed that certain changes associated with endothermic processes occurred in the thermogravimetric (TG) curve with the addition of silver nanoparticles. Hence, in the 450–600 °C interval, exothermic effects generated by the combustion of BC were observed (see Figure 4a), while in the 600–700 °C interval, it can be assumed that the oxidation of silver nanoparticles and dehydroxylation of HAp occurred [15].

We observed (Figure 4) significant differences regarding mass loss between the samples obtained by in situ vs. ex situ method. The total weight loss in the temperature range of 30–700 °C was 45% for BC_1_, 65% for BC_4_, 68% for BC_5_, and 70% for BC_8_. The composites obtained in situ had a lower weight loss, which suggests good loading of BC with calcium phosphate phases (HAp).

**Figure 5:**
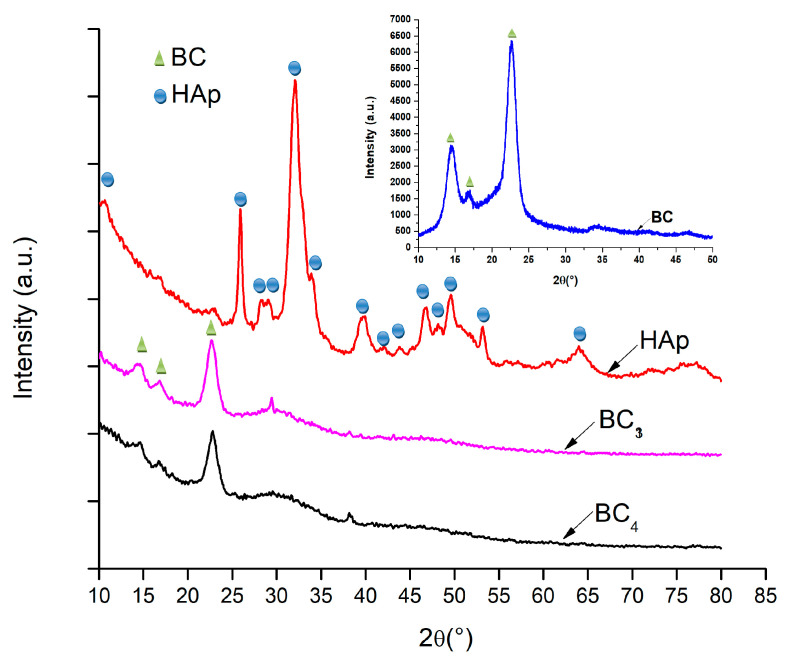
XRD analysis corresponding to the composites samples BC_3_ and BC_4,_ and hydroxyapatite (HAp) obtained by the coprecipitation method.

In order to demonstrate the composition, hydroxyapatite was analyzed by XRD technique. The diffractograms are presented in Figure 5.

Due to the fact that BC_5_–BC_8_ samples were made by direct mixing of bacterial cellulose with hydroxyapatite (ex situ), the composition of this sample was not expected to change; therefore, the XRD analysis was only performed for bacterial cellulose, simple hydroxyapatite, and BC_3_ and BC_4_ composites (in situ) [44,45].

It was observed that, in all the analyzed samples, the existence of bacterial cellulose and HAp was obvious. In addition, the low-intensity peak around 2θ = 38°, which can be assigned to the (111) crystalline plane, indicated the presence of silver nanoparticles in the composite structure. Investigation of the composites BC_3_ and BC_4_ revealed peaks located at 2θ values of 15, 16, and 23°, which can be attributed to bacterial cellulose according to ICDD 00-056-1718.

**Figure 6:**
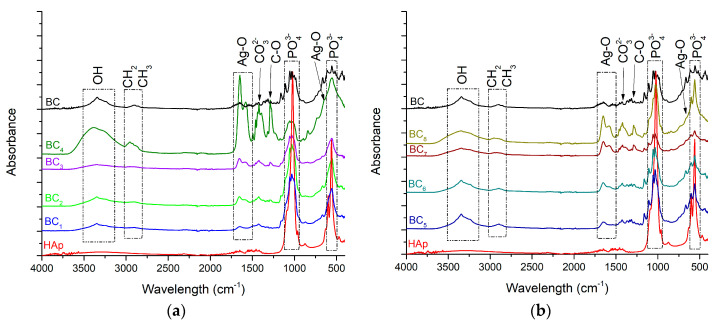
FT-IR analysis corresponding to the (**a**) in situ and (**b**) ex-situ composite samples.

As the HAp peaks were poorly visible in XRD analysis, FT-IR analyses were used to better highlight hydroxyapatite formation. The results are presented in Figure 6.

The vibrational frequencies characteristic of bacterial cellulose were observed at 3500–3200 cm^−1^ (OH stretch vibrations) and 2958 cm^−1^ (CH_2_ and CH_3_ stretch vibrations). The wide band observed in the region of 3500–3200 cm^−1^, attributed to the hydroxyl groups within the bacterial cellulose, increased in absorbance with higher silver content. This behavior suggested that the presence of HAp crystals affected cellulose hydroxyl groups, probably by covering them at the surface. Furthermore, the change observed for the band attributed to intramolecular hydrogen bonding (~3500 cm^−1^) confirmed a strong interaction between the OH groups and calcium phosphate. The chemical interaction between HAp and BC stabilized the composite so that it could maintain its mechanical integrity, an aspect required for bone substituents [46].

The FT-IR bands observed at 1020 cm^−1^ and 570–600 cm^−1^ were attributed to the vibrational modes of PO_4_^3−^. Because the stretching vibration of CO_3_^2−^ also appeared (at 1418 cm^−1^), absorption of CO_2_ from the air is suggested [47]. This is mainly a result of the affinity for carbonate of HAp as well as the lack of heat treatment during the in situ synthesis (which favors the release of CO_2_). Carbonated hydroxyapatite contributes to the biomimetism increase of the obtained composites, which can promote the process of osteoregeneration. It was observed that the in situ method accelerated the nucleation of HAp crystals onto BC fibers instead of crystallization as higher absorbance values were registered for BC_5_–BC_8_ samples, which contained highly crystalized HAp. The bands observed at 1641 and 643 cm^−1^ correspond to the stretching and deformation vibrations of AgO, respectively, thus confirming the presence of silver in the obtained materials [48]. This result supports the idea that the composite material developed here possesses essential physicochemical properties and could be very useful for biomedical applications, especially hard tissue engineering.

Through the two analyses performed, it was possible to notice the elemental composition of materials (EDS) as well as the homogeneity of hydroxyapatite particle dispersion.

**Figure 7:**
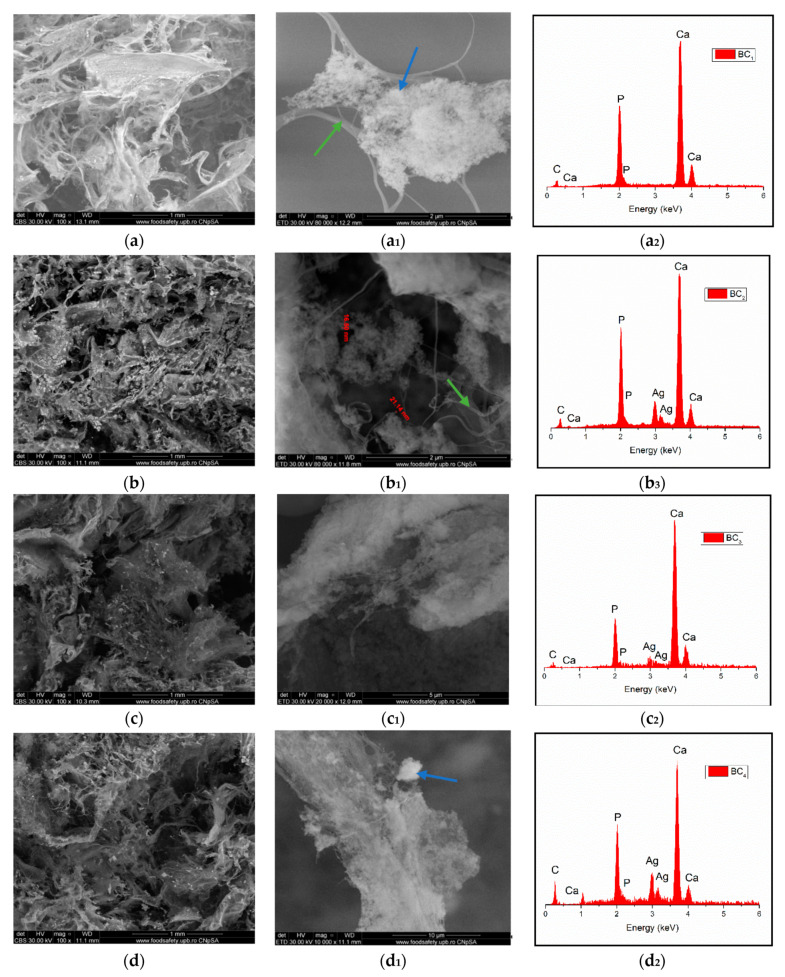
High-resolution backscattered-electron (BSE) images (and EDS spectra at 100× magnification for: BC_1_ (**a**,**a_1_**,**a_2_**), BC_2_ (**b**,**b_1_**,**b_2_**), BC_3_ (**c**,**c_1_**,**c_2_**), BC_4_ (**d**,**d_1_**,**d_2_**). (where **a_1_**–**d_1_** images represent a high magnification of a-d images area; green arrow indicates the fibrous structure of BC and blue arrow indicate inorganic particles).

**Figure 8:**
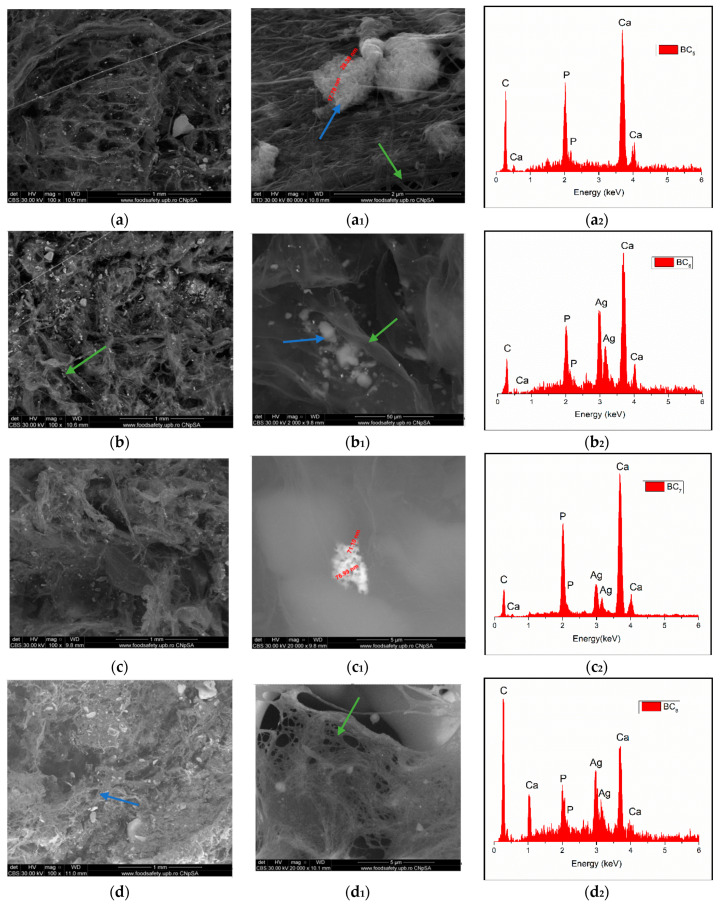
High-resolution backscattered electron (BSE) images and EDS spectra at 100× magnification for BC_5_ (**a**,**a_1_**,**a_2_**), BC_6_ (**b**,**b_1_**,**b_2_**), BC_7_ (**c**,**c_1_**,**c_2_**), and BC_8_ (**d**,**d_1_**,**d_2_**) (where **a_1_**–**d_1_** images represent a high magnification of a–d image area; green arrow indicates the fibrous structure of BC and blue arrow indicate inorganic particles).

The SEM image highlighted the fibrous structure of BC (see green arrow), which were decorated with inorganic particles (see blue arrow). In the SEM images performed on the composites in which HAp was obtained in situ (Figure 7a–d), a better homogeneity was observed compared to the cases in which HAp was obtained separately and subsequently mixed with BC (ex situ) (Figure 8a–d. The interaction between HAp nanoparticles distributed in the 3D network of BC stabilized the composite so that it could maintain its mechanical integrity, an aspect required for bone substituents. In addition, EDS analysis confirmed the presence of the elements specific for hydroxyapatite (Ca, P, and O) as well as the presence of silver for the samples in which it was added (Figure 7(b_2_–d_2_) and Figure 8(b_2_–d_2_)).

**Figure 9:**
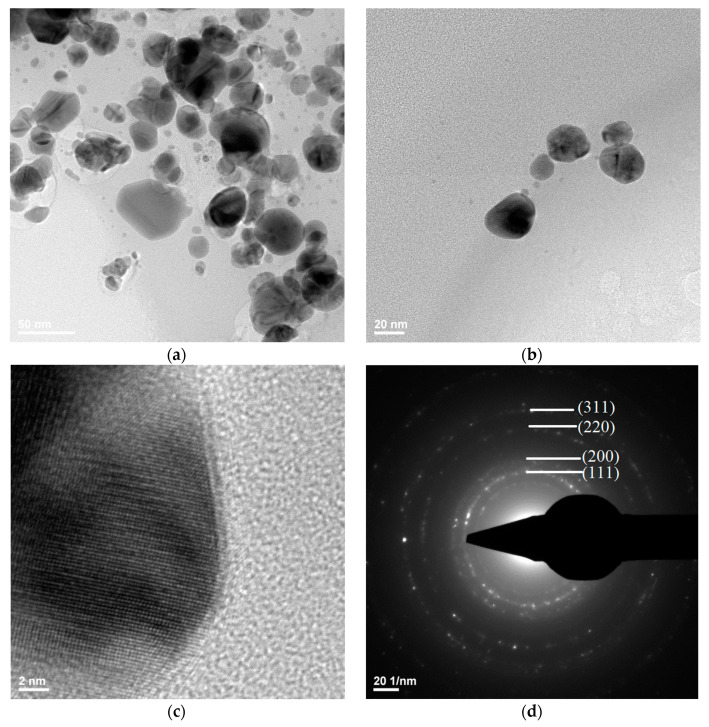
TEM (**a**,**b**), HR-TEM (**c**), and SAED (**d**) images for silver.

Transmission electron microscopy images showed the silver nanostructure (Figure 9a,b), with the dimensions of the silver particles being in the range 3–60 nm. It could be observed that the quasi-spherical morphology of nanosilver and some areas were darker while others were brighter; the darker areas indicate a higher degree of crystallinity of the material.

Figure 9d shows a SAED image with information on the crystallinity of the analyzed material. The presence of diffraction rings with higher light intensity shows a high degree of crystallinity.

**Figure 10:**
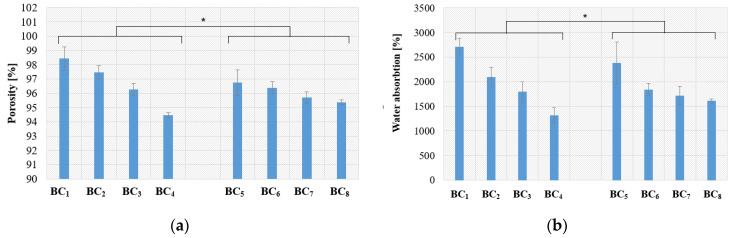
Porosity (**a**) and water absorption (**b**) results for the obtained composites (presented as mean ± S.D. of three replicates and * *p* < 0.005 obtained by single-factor ANOVA test.

The calculated open porosity for each prepared material is presented in Figure 10a, and the calculated water absorption is presented in Figure 9b.

Figure 10a shows that the composites obtained by in situ approach had a large porosity compared to samples obtained by ex situ. This suggests that the in situ route will provide a biodegradable polymer with excellent water retention and, possibly, good osteoinductivity, which can be used as an artificial substitute for hard tissue.

As can be observed, when the silver nanoparticle concentration increased, the composite porosity decreased for susceptible types of composite, which resulted in lower absorption capacity. Even though high porosity, which is associated with increased absorption capacity, is an important structural parameter for bone substituents, the registered decrease due to Ag addition is not significant in this case as the water absorption was still greater than 1000–1500%.

**Figure 11:**
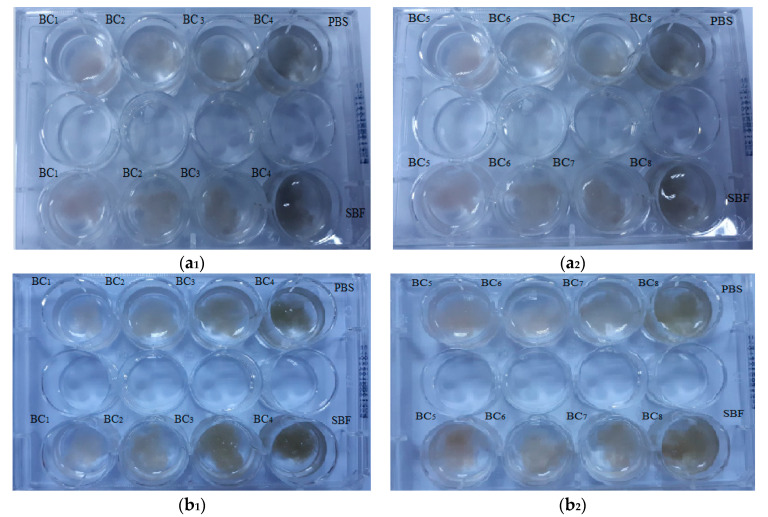
Macroscopic pictures of degradation of BC composites initially (**a_1_**,**a_2_**) and after seven days of immersion in phosphate-buffered saline (PBS) and simulated body fluid (SBF) (**b_1_**,**b_2_**).

Visual inspection (Figure 11) is an efficient technique for investigation of simulated in vitro degradation of BC samples, and it has also been used in recent literature [49] in order to investigate the degradation of cellulose-based materials. Generally, visual inspection implies macroscopic pictures of the immersed samples while observing, in a qualitative manner, the presence of detached fragments, the apparition of denser fragments that may provide additional mechanical integrity for cell growth, and so on. As expected, the composite materials did not show major changes after immersion in PBS and SBF.

**Figure 12:**
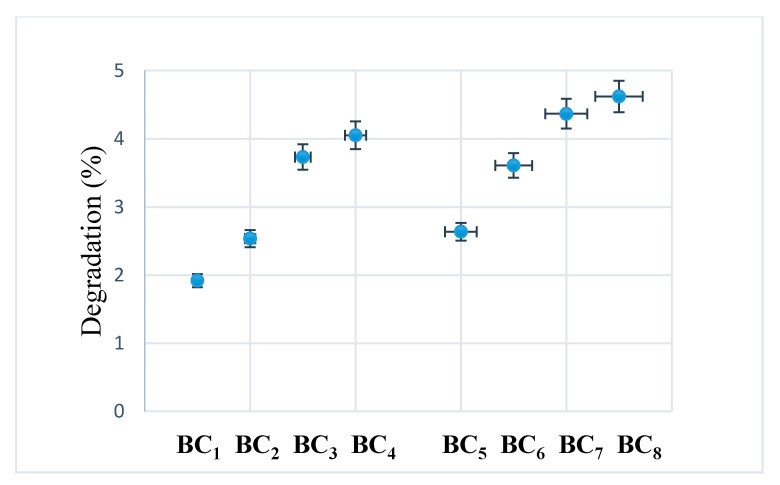
Degradability of composites after seven days of immersion in SBF at room temperature.

After seven days, no major visual changes were observed, and the degradation was below 5% (see Figure 12), a sign that bacterial cellulose prevented the disintegration of the composite. A rapid biodegradation of the implanted material is not desired because it takes time for it to integrate better into the host tissue. Another problem is that by biodegradation, remnants/fragments of the material can reach the level of sensitive areas, which would be fatal.

Due to the higher homogeneity and better interactions between the phosphate phase (HAp) and BC, the composites obtained in situ had a lower degradation compared to those obtained using ex situ methods.

### Antimicrobial Potential

**Figure 13:**
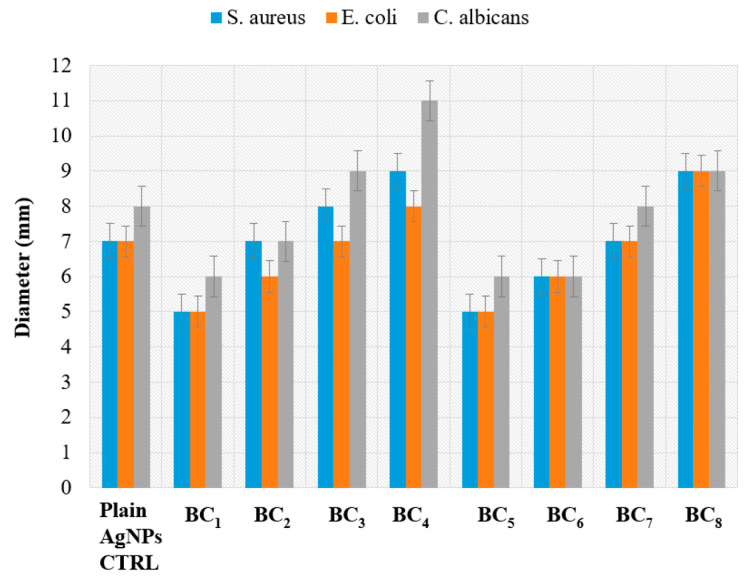
The diameter of the growth inhibition zone of the tested microorganisms grown in the presence of the obtained BC samples containing various amounts of AgNPs. The plain AgNP control is represented by 10 uL of AgNPs (used at maximum equivalent amount contained in BC samples), which was added to a commercial filter paper disc of a similar size as the obtained BC samples.

The antimicrobial effect of the obtained composite materials was different among the tested samples, being influenced by the synthesis approach, AgNP content, and microbial species. Figure 13 shows the diameters of the inhibition area of the microorganisms grown in the presence of the tested materials.

Because composites BC_1_ and BC_5_ did not contain antibacterial AgNPs, they were used as control samples for evaluation of the other samples.

The antimicrobial characteristic of the obtained composites was clearly influenced by the concentration of AgNPs, with samples with higher content of silver exhibiting the greatest microbial growth inhibition, regardless of the synthesis approach. It was observed that the materials obtained by the in situ method had a more pronounced overall antibacterial characteristic (growth inhibition zones ranging 5–11 mm) compared to the samples obtained by the ex situ method (diameter of inhibition zones ranging 5–9 mm).

The different antimicrobial effects of the materials obtained by in situ and ex situ routes correlated with their physicochemical properties. Samples obtained by the in situ route showed a larger porosity, suggesting that the bioactive compounds (i.e., AgNPs) may be absorbed more efficiently in pores. Moreover, a higher porosity degree can be directly associated with an easier release of the bioactive agent, therefore inducing an increase in the antimicrobial activity of the final composite. This idea is supported by the results obtained with the control AgNPs utilized in equivalent amounts and added to sterile commercial filter paper, which demonstrated lower inhibition zones.

The most efficient growth inhibition was observed against the yeast strain *C. albicans* ATCC 10231, with the result being relevant for samples obtained by both in situ and ex situ methods. However, the composites obtained in situ also showed increased antimicrobial activity against the Gram-positive *S. aureus* strain. This result suggests that the obtained composite materials may act differently on microbial cells, depending on the particularities of their cellular wall. Such differences were observed before with silver nanoparticles [50,51,52,53].

## 5. Conclusions

In this study, we report the synthesis of hydroxyapatite–bacterial cellulose silver nanocomposites obtained by two routes using coprecipitation, namely, in situ and ex situ assembly. These materials contained an organic part (bacterial cellulose), an inorganic part (hydroxyapatite), and an antimicrobial agent (AgNPs) contained in various amounts, thereby conferring new bioactive properties on the composite materials. Physicochemical and antimicrobial studies demonstrated that the most efficient in terms of potential biomedical applications were the samples obtained by the in situ approach. The porosity range of the in situ materials was greater than the porosity of ex situ composites, while the best antimicrobial activity was observed for the material coded BC_4_, which had a content of 5 wt % AgNPs. Due to the physicochemical structure, together with the already demonstrated great antimicrobial properties and low biodegradability of these materials, they have potential applications as successful candidates for biomedical applications, especially in hard tissue engineering. Their current limitation relates to the fact that further tests performed on osteoblast differentiation and mineralization (e.g., alkaline phosphatase and alizarin red S) are needed.

## Figures and Tables

**Table 1:** Bacterial cellulose, HAp, and AgNP content in the final composites.

Sample Name	Bacterial Cellulose Content (%)	HAp Content (%)	AgNP Content (%)
BC_1_	50	50	0
BC_2_	50	49	1
BC_3_	50	48	2
BC_4_	50	50	5
BC_5_	50	50	0
BC_6_	50	49	1
BC_7_	50	48	2
BC_8_	50	45	5

## References

[1] Sarkar C., Chowdhuri A.R., Garai S., Chakraborty J., Sahu S.K. (2019). Three-dimensional cellulose-hydroxyapatite nanocomposite enriched with dexamethasone loaded metal–organic framework: A local drug delivery system for bone tissue engineering. Cellulose.

[2] Shkarina S., Shkarin R., Weinhardt V., Melnik E., Vacun G., Kluger P.J., Loza K., Epple M., Ivlev S.I., Baumbach T. (2018). 3D biodegradable scaffolds of polycaprolactone with silicate-containing hydroxyapatite microparticles for bone tissue engineering: High-resolution tomography and in vitro study. Sci. Rep..

[3] Atila D., Karataş A., Evcin A., Keskin D., Tezcaner A. (2019). Bacterial cellulose-reinforced boron-doped hydroxyapatite/gelatin scaffolds for bone tissue engineering. Cellulose.

[4] Blanco Parte F.G., Santoso S.P., Chou C.-C., Verma V., Wang H.-T., Ismadji S., Cheng K.-C. (2020). Current progress on the production, modification, and applications of bacterial cellulose. Crit. Rev. Biotechnol..

[5] Woltje M., Ostermann K., Aibibu D., Rodel G., Cherif C. (2019). Session 12: Biomaterials. Biomed. Tech..

[6] Lu T., Feng S., He F., Ye J. (2020). Enhanced osteogenesis of honeycomb β-tricalcium phosphate scaffold by construction of interconnected pore structure: An in vivo study. J. Biomed. Mater. Res. Part A.

[7] Hapuhinna K., Gunaratne R., Pitawala J. (2019). Comparison between Differently Synthesized Hydroxyapatite Composites for Orthopedic Applications. J. Mater. Sci. Chem. Eng..

[8] Arcos D., Vallet-Regí M. (2020). Substituted hydroxyapatite coatings of bone implants. J. Mater. Chem. B.

[9] Chang H.-H., Yeh C.-L., Wang Y.-L., Fu K.-K., Tsai S.-J., Yang J.-H., Lin C.-P. (2020). Neutralized Dicalcium Phosphate and Hydroxyapatite Biphasic Bioceramics Promote Bone Regeneration in Critical Peri-Implant Bone Defects. Materials.

[10] Liu Y., Gu J., Fan D. (2020). Fabrication of High-Strength and Porous Hybrid Scaffolds Based on Nano-Hydroxyapatite and Human-Like Collagen for Bone Tissue Regeneration. Polymers.

[11] Nicomrat D. (2020). Silver Nanoparticles Impregnated Biocellulose Produced by Sweet Glutinous Rice Fermentation with the Genus Acetobacter. E3S Web Conf..

[12] Maneerung T., Tokura S., Rujiravanit R. (2008). Impregnation of silver nanoparticles into bacterial cellulose for antimicrobial wound dressing. Carbohydr. Polym..

[13] Velu R., Calais T., Jayakumar A., Raspall F. (2019). A Comprehensive Review on Bio-Nanomaterials for Medical Implants and Feasibility Studies on Fabrication of Such Implants by Additive Manufacturing Technique. Materials.

[14] Busuioc C., Stroescu M., Stoica-Guzun A., Voicu G., Jinga S.-I. (2016). Fabrication of 3D calcium phosphates based scaffolds using bacterial cellulose as template. Ceram. Int..

[15] Draghici A.-D., Busuioc C., Mocanu A., Nicoara A.-I., Iordache F., Jinga S.-I. (2020). Composite scaffolds based on calcium phosphates and barium titanate obtained through bacterial cellulose templated synthesis. Mater. Sci. Eng. C.

[16] Khan F., Dahman Y. (2012). A novel approach for the utilization of biocellulose nanofibres in polyurethane nanocomposites for potential applications in bone tissue implants. Des. Monomers Polym..

[17] Busuioc C., Ghitulica C.D., Stoica A., Stroescu M., Voicu G., Ionita V., Averous L., Jinga S.I. (2018). Calcium phosphates grown on bacterial cellulose template. Ceram. Int..

[18] Halib N., Ahmad I., Grassi M., Grassi G. (2019). The remarkable three-dimensional network structure of bacterial cellulose for tissue engineering applications. Int. J. Pharm..

[19] Frone A.N., Panaitescu D.M., Nicolae C.A., Gabor A.R., Trusca R., Casarica A., Stanescu P.O., Baciu D.D., Salageanu A. (2020). Bacterial cellulose sponges obtained with green cross-linkers for tissue engineering. Mater. Sci. Eng. C.

[20] Liyaskina E., Revin V., Paramonova E., Nazarkina M., Pestov N., Revina N., Kolesnikova S. (2017). Nanomaterials from bacterial cellulose for antimicrobial wound dressing. Journal of Physics: Conference Series.

[21] Mocanu A., Isopencu G., Busuioc C., Popa O.-M., Dietrich P., Socaciu-Siebert L. (2019). Bacterial cellulose films with ZnO nanoparticles and propolis extracts: Synergistic antimicrobial effect. Sci. Rep..

[22] Hodel K.V.S., Fonseca L.M.d.S., Santos I.M.d.S., Cerqueira J.C., Santos-Júnior R.E.d., Nunes S.B., Barbosa J.D.V., Machado B.A.S. (2020). Evaluation of Different Methods for Cultivating Gluconacetobacter hansenii for Bacterial Cellulose and Montmorillonite Biocomposite Production: Wound-Dressing Applications. Polymers.

[23] Basu P., Saha N., Alexandrova R., Saha P. (2019). Calcium Phosphate Incorporated Bacterial Cellulose-Polyvinylpyrrolidone Based Hydrogel Scaffold: Structural Property and Cell Viability Study for Bone Regeneration Application. Polymers.

[24] Eslahi N., Mahmoodi A., Mahmoudi N., Zandi N., Simchi A. (2020). Processing and properties of nanofibrous bacterial cellulose-containing polymer composites: A review of recent advances for biomedical applications. Polym. Rev..

[25] Chiaoprakobkij N., Seetabhawang S., Sanchavanakit N., Phisalaphong M. (2019). Fabrication andcharacterization of novel bacterial cellulose/alginate/gelatin biocomposite film. J. Biomater. Sci. Polym. Ed..

[26] Bodea I.M., Cătunescu G.M., Stroe T.F., Dîrlea S.A., Beteg F.I. (2020). Applications of bacterial-synthesized cellulose in veterinary medicine—A review. Acta Vet. Brno.

[27] Ou Q., Huang K., Fu C., Huang C., Fang Y., Gu Z., Wu J., Wang Y. (2020). Nanosilver-incorporated halloysite nanotubes/gelatin methacrylate hybrid hydrogel with osteoimmunomodulatory and antibacterial activity for bone regeneration. Chem. Eng. J..

[28] Hussain Z., Abourehab M.A., Khan S., Thu H.E. (2020). Silver nanoparticles: A promising nanoplatform for targeted delivery of therapeutics and optimized therapeutic efficacy. Metal Nanoparticles for Drug Delivery and Diagnostic Applications.

[29] Khurshid Z., Zafar M.S., Hussain S., Fareed A., Yousaf S., Sefat F. (2020). Silver-substituted hydroxyapatite. Handbook of Ionic Substituted Hydroxyapatites.

[30] Preethi G.U., Unnikrishnan B.S., Sreekutty J., Archana M.G., Anupama M.S., Shiji R., Raveendran Pillai K., Joseph M.M., Syama H.P., Sreelekha T.T. (2020). Semi-interpenetrating nanosilver doped polysaccharide hydrogel scaffolds for cutaneous wound healing. Int. J. Biol. Macromol..

[31] Janarthanan P., Sathasivam T., Li T.H., Dahlan N.A., Paramasivam R. (2020). Silver Nanoparticles: Biological Synthesis and Applications. Biological Synthesis of Nanoparticles and Their Applications.

[32] Ali G., Abd El-Moez S., Abdel-Fattah W. (2019). Synthesis and characterization of nontoxic silvernano-particles with preferential bactericidal activity. Biointerface Res. Appl. Chem..

[33] Wijnhoven S.W.P., Peijnenburg W.J.G.M., Herberts C.A., Hagens W.I., Oomen A.G., Heugens E.H.W., Roszek B., Bisschops J., Gosens I., Van De Meent D. (2009). Nano-silver—A review of available data and knowledge gaps in human and environmental risk assessment. Nanotoxicology.

[34] Khatoon Z., McTiernan C.D., Suuronen E.J., Mah T.-F., Alarcon E.I. (2018). Bacterial biofilm formation on implantable devices and approaches to its treatment and prevention. Heliyon.

[35] Zimmermann K.A., LeBlanc J.M., Sheets K.T., Fox R.W., Gatenholm P. (2011). Biomimetic design of a bacterial cellulose/hydroxyapatite nanocomposite for bone healing applications. Mater. Sci. Eng. C.

[36] Saska S., Barud H., Gaspar A., Marchetto R., Ribeiro S.J.L., Messaddeq Y. (2011). Bacterial cellulose-hydroxyapatite nanocomposites for bone regeneration. Int. J. Biomater..

[37] Wan Y., Huang Y., Yuan C., Raman S., Zhu Y., Jiang H., He F., Gao C. (2007). Biomimetic synthesis of hydroxyapatite/bacterial cellulose nanocomposites for biomedical applications. Mater. Sci. Eng. C.

[38] Hong L., Wang Y.L., Jia S.R., Huang Y., Gao C., Wan Y.Z. (2006). Hydroxyapatite/bacterial cellulose composites synthesized via a biomimetic route. Mater. Lett..

[39] Wan Y.Z., Hong L., Jia S.R., Huang Y., Zhu Y., Wang Y.L., Jiang H.J. (2006). Synthesis and characterization of hydroxyapatite–bacterial cellulose nanocomposites. Compos. Sci. Technol..

[40] Grande C.J., Torres F.G., Gomez C.M., Carmen Bañó M. (2009). Nanocomposites of bacterial cellulose/hydroxyapatite for biomedical applications. Acta Biomater..

[41] Dincă V., Mocanu A., Isopencu G., Busuioc C., Brajnicov S., Vlad A., Icriverzi M., Roseanu A., Dinescu M., Stroescu M. (2020). Biocompatible pure ZnO nanoparticles-3D bacterial cellulose biointerfaces with antibacterial properties. Arab. J. Chem..

[42] Leau S.-A., Marin Ş., Coară G., Albu L., Constantinescu R.R., Kaya M.A., Neacşu I.-A. Study of wound-dressing materials based on collagen, sodium carboxymethylcellulose and silver nanoparticles used for their antibacterial activity in burn injuriess. Proceedings of the International Conference on Advanced Materials and Systems (ICAMS).

[43] Ghorbani F., Li D., Ni S., Zhou Y., Yu B. (2020). 3D printing of acellular scaffolds for bone defect regeneration: A review. Mater. Today Commun..

[44] Anghel I., Grumezescu A.M., Holban A.M., Gheorghe I., Vlad M., Anghel G.A., Balaure P.C., Chifiriuc C.M., Ciuca I.M. (2014). Improved activity of aminoglycosides entrapped in silica networks against microbial strains isolated from otolaryngological infections. Farmacia.

[45] Stumpf T.R., Yang X., Zhang J., Cao X. (2018). In situ and ex situ modifications of bacterial cellulose for applications in tissue engineering. Mater. Sci. Eng. C.

[46] Yin N., Chen S.-Y., Ouyang Y., Tang L., Yang J.-X., Wang H.-P. (2011). Biomimetic mineralization synthesis of hydroxyapatite bacterial cellulose nanocomposites. Progress Nat. Sci. Mater. Int..

[47] Marković S., Veselinović L., Lukić M.J., Karanović L., Bračko I., Ignjatović N., Uskoković D. (2011). Synthetical bone-like and biological hydroxyapatites: A comparative study of crystal structure and morphology. Biomed. Mater..

[48] Markovic M., Fowler B.O., Tung M.S. (2004). Preparation and comprehensive characterization of a calcium hydroxyapatite reference material. J. Res. Natl. Inst. Stand. Technol..

[49] Suk J.S., Xu Q., Kim N., Hanes J., Ensign L.M. (2016). PEGylation as a strategy for improving nanoparticle-based drug and gene delivery. Adv. Drug Deliv. Rev..

[50] Hu Y., Catchmark J.M. (2011). In vitro biodegradability and mechanical properties of bioabsorbable bacterial cellulose incorporating cellulases. Acta Biomater..

[51] Abbaszadegan A., Ghahramani Y., Gholami A., Hemmateenejad B., Dorostkar S., Nabavizadeh M., Sharghi H. (2015). The effect of charge at the surface of silver nanoparticles on antimicrobial activity against gram-positive and gram-negative bacteria: A preliminary study. J. Nanomater..

[52] Ayodele A.T., Valizadeh A., Adabi M., Esnaashari S.S., Madani F., Khosravani M., Adabi M. (2017). Ultrasound nanobubbles and their applications as theranostic agents in cancer therapy: A review. Biointerface Res. Appl. Chem..

[53] Sabry N.M., Tolba S., Abdel-Gawad F.K., Bassem S.M., Nassar H.F., El-Taweel G.E., Okasha A., Ibrahim M. (2018). Interaction between nano silver and bacteria: Modeling approach. Biointerface Res. Appl. Chem..

